# The COVID-19 pandemic impact on pediatric surgery residency programs

**DOI:** 10.1016/j.heliyon.2021.e07199

**Published:** 2021-06-01

**Authors:** Gunadi, Naisya Balela, Alvin Santoso Kalim, William Widitjiarso, Fadil Fahri, Audric Kenny Tedja, Eko Purnomo, Andi Dwihantoro, Nunik Agustriani, Akhmad Makhmudi

**Affiliations:** a Pediatric Surgery Division, Department of Surgery, Faculty of Medicine, Public Health and Nursing, Universitas Gadjah Mada/Dr. Sardjito Hospital, Yogyakarta 55281, Indonesia; b Pediatric Surgery Division, Department of Surgery, Faculty of Medicine, Public Health and Nursing, Universitas Gadjah Mada/UGM Academic Hospital, Yogyakarta 55291, Indonesia; c Pediatric Surgery Division, Department of Surgery, Faculty of Medicine, Public Health and Nursing, Universitas Gadjah Mada/Faculty of Medicine, Universitas Sebelas Maret, Surakarta 57126, Indonesia

**Keywords:** COVID-19 pandemic, Pediatric surgery, Residency program

## Abstract

**Background:**

The residency program as a part of the clinical services itself has been influenced by the COVID-19 outbreak. Several reports have been published regarding the impact of COVID-19 on the residency programs; however, all studies were performed in developed countries or did not comprehensively analyze what residents think about the COVID-19 impact on their residency program. We investigated the impact of the COVID-19 pandemic on the pediatric surgery residency program in our institution as an important part of hospital medical services.

**Methods:**

We developed and distributed a questionnaire to pediatric surgery residents in our institution who were registered from January 2015–July 2020. The questionnaire was consisting of 24 questions: a) the perspectives of residents about COVID-19 infection during their residency program; b) the learning process; c) academic evaluations; and d) residents' suggestions to improve the quality of their residency program during the outbreak.

**Results:**

Most (82.6%) pediatric surgery residents agreed that elective surgeries should be postponed during the pandemic. Before the outbreak, almost all (82.6%) residents used textbooks and journals as their primary sources of learning, while during the outbreak, 69.5% of residents shifted to use online lectures either from the school or Association of Pediatric Surgeons. Interestingly, 91.3% of participants agreed that they had more time to complete their academic assignments during the pandemic.

**Conclusions:**

The pandemic has had a significant impact on the development of pediatric surgery residency programs. Moreover, the responses to the questionnaire are affected by the seniority and sex of the residents. A comprehensive approach is needed to maintain the high standard of competence of pediatric surgery without compromising our safety from the COVID-19 infection risk.

## 1 Introduction

Since the World Health Organization (WHO) declared COVID-19 as a worldwide pandemic on March 11, 2020 [1], clinical practices have been severely affected worldwide. The residency program as a part of the clinical services itself has also been influenced by the outbreak [2, 3, 4].

Our institution was officially established in 1949 as a national university. Considered as one of the oldest universities in our country, our institution serves as a pillar of educational training and academic awakening in our country. Now, our institution has 18 Faculties, one Postgraduate School (master's and doctoral program), one Vocational School and 20 Residency Programs, including pediatric surgery training [5]. The pediatric surgery residency program has been established in our institution since 2006 with a length of training of 5 years [6].

Several reports have been published regarding the impact of COVID-19 on the residency programs; however, all studies were performed in developed countries [2, 3] or did not comprehensively analyze what residents think about the COVID-19 impact on their residency program [4]. Moreover, there is an uncertainty when the COVID-19 pandemic will end, and the number of cases is increasing, particularly in our province of 44,746 cases and 1,183 deaths per June 1, 2021. Therefore, we aimed to investigate the impact of the COVID-19 pandemic on the residency program in our institution, particularly pediatric surgery training, from the perspectives of residents.

## 2 Material and methods

### 2.1 Questionnaire

We developed and distributed a questionnaire to 23 pediatric surgery residents in our institution during December 2020. Twenty-three residents were registered from January 2015–July 2020. The questionnaire was developed by educators/attending pediatric surgeons. The educators/attending pediatric surgeons convened to designing the questions. The questionnaire consisted of 24 questions concerning: a) the perspectives of residents about COVID-19 infection during their residency program (n = 5); b) the learning process during the outbreak (n = 12); c) academic evaluations (n = 6); and d) the residents' suggestions to improve the quality of their residency program during the outbreak (n = 1, open question) (Table 1).

**Table 1:** Questionnaire to evaluate the pediatric surgery residency program in our institution.

No	Question
	**A. Perspective resident on COVID-19 infection during the residency program**
1	Do you feel worried about getting COVID-19 infection during performing surgeries?
	a. Yes
	b. No
	c. Don't know
2	In your opinion, do elective pediatric surgical procedures need to be reduced during the COVID-19 pandemic?
	a. Disagree
	b. Neutral
	c. Agree
3	During COVID-19, what is the percentage of decrease in the number of the elective pediatric surgeries?
	a. None
	b. <25%
	c. 25-<50%
	d. 50-<75%
	e. 75-100%
4	During COVID-19, what is the percentage of decrease in the number of the emergency pediatric surgeries?
	a. None
	b. <25%
	c. 25-<50%
	d. 50-<75%
	e. 75-100%
5	The compliance level of resident in following the guidelines of personal protective equipment use in the hospital during the pandemic is low.
	a. Disagree
	b. Neutral
	c. Agree
	**B. Learning process during the outbreak**
6	Which learning methods did you usually use before COVID-19 outbreak?
	a. Textbooks and journals
	b. Webinar, lectures and workshops held by the school or Indonesian Association of Pediatric Surgeon (PERBANI) or other Pediatric Surgery Association
	c. Virtual didactic tools (example: Virtual Reality Anatomy, Minimally Invasive Surgery Trainer-Virtual Reality, etc.)
	d. Others
7	Which learning methods do you usually use during the outbreak?
	a. Textbooks and journals
	b. Webinar, lectures and workshops held by the school or Indonesian Association of Pediatric Surgeon (PERBANI) or other Pediatric Surgery Association
	c. Virtual didactic tools (example: Virtual Reality Anatomy, Minimally Invasive Surgery Trainer-Virtual Reality, etc.)
	d. Others
8	During the pandemic, the online morning report is already good to achieve the skills and knowledge.
	a. Disagree
	b. Neutral
	c. Agree
9	Offline morning report is better than online meeting to achieve the skills and knowledge.
	a. Disagree
	b. Neutral
	c. Agree
10	The frequency of morning report should be increased during the pandemic.
	a. Disagree
	b. Neutral
	c. Agree
11	Live view surgery method is important to gain the surgical skill during COVID-19 pandemic.
	a. Disagree
	b. Neutral
	c. Agree
12	Live view surgery method is important for all pediatric surgical cases, including elective and emergency cases.
	a. Disagree
	b. Neutral
	c. Agree
13	Live view surgery method is important only for interesting pediatric surgical cases.
	a. Disagree
	b. Neutral
	c. Agree
14	The restriction of resident number in the outpatients' clinics during the outbreak hampers them from gaining the necessary skills and knowledge.
	a. Disagree
	b. Neutral
	c. Agree
15	The restriction of resident number during the ward rounds inhibits them from obtaining the necessary skills and knowledge.
	a. Disagree
	b. Neutral
	c. Agree
16	Virtual outpatients and ward round method are necessary to obtain the needed skills, particularly for residents who were not on duty in the hospital.
	a. Disagree
	b. Neutral
	c. Agree
17	The competence gain is declining during the pandemic.
	a. Disagree
	b. Neutral
	c. Agree
	**C. Academic evaluation**
18	During COVID-19 pandemic, you have more time to finish the academic assignments.
	a. Disagree
	b. Neutral
	c. Agree
19	The completion of the academic assignments can be achieved on time.
	a. Disagree
	b. Neutral
	c. Agree
20	COVID-19 pandemic prolongs the study periods.
	a. Disagree
	b. Neutral
	c. Agree
21	COVID-19 pandemic hampers the completion of the thesis.
	a. Disagree
	b. Neutral
	c. Agree
22	COVID-19 pandemic inhibits the level up examination process.
	a. Disagree
	b. Neutral
	c. Agree
23	National board examination is delayed by the outbreak.
	a. Disagree
	b. Neutral
	c. Agree
	**D. Resident suggestions to improve the quality of residency program during the outbreak**
24	What are your suggestions to gain the necessary skills and knowledge of pediatric surgery during the outbreak?

The Medical and Health Research Ethics Committee of our institution approved this study (KE/FK/0718/EC/2020). Written informed consent was obtained from all participants before joining in this study.

### 2.2 Statistical test

The association between variables were determined by the Fisher-Exact test and *p*-value < 0.05 was considered as significant.

## 3 Results

### 3.1 Baseline characteristics

The total number of pediatric surgery residents was 23, consisting of 15 males and 8 females (Table 2). All residents (100%) completely responded to the questionnaire.

**Table 2:** Baseline characteristics of pediatric surgery residents in our institution.

Characteristic	N (%)
Sex	
Male	15 (65.2)
Female	8 (34.8)
Pediatric surgery competence classification	
Junior	8 (34.8)
Middle	6 (26.1)
Senior	9 (39.1)

### 3.2 Perspectives of residents concerning COVID-19 infection during the residency program

Most pediatric surgery residents (82.6%) agreed that elective surgeries should be postponed during the pandemic because they (100%) worried about getting infected with SARS-Cov-2 during the surgical procedures. Most of them (78.2% and 86.9%, respectively) thought that the number of elective and emergency surgeries decreased by approximately 25-<75% and 25-<50%, respectively, during the pandemic (Table 3).

**Table 3:** Resident responses on the questionnaire concerning the effect of the COVID-19 pandemic on the pediatric surgery residency program.

No	Question	N (%)
	**A. Perspective resident on COVID-19 infection during the residency program**	
1	Do you feel worried about getting COVID-19 infection during performing surgeries?	
	a. Yes	23 (100)
	b. No	0
	c. Don't know	0
2	In your opinion, do elective pediatric surgical procedures need to be reduced during the COVID-19 pandemic?	
	a. Disagree	2 (8.7)
	b. Neutral	2 (8.7)
	c. Agree	19 (82.6)
3	During COVID-19, what is the percentage of decrease in the number of the elective pediatric surgeries?	
	a. None	1 (4.3)
	b. <25%	3 (13.1)
	c. 25-<50%	9 (39.1)
	d. 50-<75%	9 (39.1)
	e. 75–100%	1 (4.3)
4	During COVID-19, what is the percentage of decrease in the number of the emergency pediatric surgeries?	
	a. None	0
	b. <25%	4 (17.4)
	c. 25-<50%	16 (69.5)
	d. 50-<75%	3 (13.1)
	e. 75–100%	0
5	The compliance level of resident in following the guidelines of personal protective equipment use in the hospital during the pandemic is low.	
	a. Disagree	11 (47.8)
	b. Neutral	11 (47.8)
	c. Agree	1 (4.4)
	**B. Learning process during the outbreak**	
6	Which learning methods did you usually use before COVID-19 outbreak?	
	a. Textbooks and journals	19 (82.6)
	b. Webinar, lectures and workshops held by the school or Indonesian Association of Pediatric Surgeon (PERBANI) or other Pediatric Surgery Association	3 (13.1)
	c. Virtual didactic tools (example: Virtual Reality Anatomy, Minimally Invasive Surgery Trainer-Virtual Reality, etc.)	1 (4.3)
	d. Others	0
7	Which learning methods do you usually use during the outbreak?	
	a. Textbooks and journals	6 (26.1)
	b. Webinar, lectures and workshops held by the school or Indonesian Association of Pediatric Surgeon (PERBANI) or other Pediatric Surgery Association	16 (69.5)
	c. Virtual didactic tools (example: Virtual Reality Anatomy, Minimally Invasive Surgery Trainer-Virtual Reality, etc.)	1 (4.3)
	d. Others	0
8	During the pandemic, the online morning report is already good to achieve the skills and knowledge.	
	a. Disagree	1 (4.3)
	b. Neutral	3 (13)
	c. Agree	19 (82.6)
9	Offline morning report is better than online meeting to achieve the skills and knowledge.	
	a. Disagree	5 (21.8)
	b. Neutral	3 (13)
	c. Agree	15 (65.2)
10	The frequency of morning report should be increased during the pandemic.	
	a. Disagree	4 (17.4)
	b. Neutral	12 (52.2)
	c. Agree	7 (30.4)
11	Live view surgery method is important to gain the surgical skill during COVID-19 pandemic.	
	a. Disagree	2 (8.7)
	b. Neutral	0
	c. Agree	21 (91.3)
12	Live view surgery method is important for all pediatric surgical cases, including elective and emergency cases.	
	a. Disagree	2 (8.7)
	b. Neutral	2 (8.7)
	c. Agree	19 (82.6)
13	Live view surgery method is important only for interesting pediatric surgical cases.	
	a. Disagree	5 (21.7)
	b. Neutral	0
	c. Agree	18 (78.3)
14	The restriction of resident number in the outpatients' clinics during the outbreak hampers them from gaining the necessary skills and knowledge.	
	a. Disagree	17 (73.9)
	b. Neutral	4 (17.4)
	c. Agree	2 (8.7)
15	The restriction of resident number during the ward rounds inhibits them from obtaining the necessary skills and knowledge.	
	a. Disagree	10 (43.5)
	b. Neutral	4 (17.4)
	c. Agree	9 (39.1)
16	Virtual outpatients and ward round method are necessary to obtain the needed skills, particularly for residents who were not on duty in the hospital.	
	a. Disagree	9 (39.1)
	b. Neutral	4 (17.4)
	c. Agree	10 (43.5)
17	The competence gain is declining during the pandemic.	
	a. Disagree	2 (8.7)
	b. Neutral	2 (8.7)
	c. Agree	19 (82.6)
	**C. Academic evaluation**	
18	During COVID-19 pandemic, you have more time to finish the academic assignments.	
	a. Disagree	1 (4.3)
	b. Neutral	1 (4.3)
	c. Agree	21 (91.3)
19	The completion of the academic assignments can be achieved on time.	
	a. Disagree	1 (4.3)
	b. Neutral	2 (8.7)
	c. Agree	20 (87)
20	COVID-19 pandemic prolongs the study periods.	
	a. Disagree	10 (43.5)
	b. Neutral	6 (26.1)
	c. Agree	7 (30.4)
21	COVID-19 pandemic hampers the completion of the thesis.	
	a. Disagree	10 (43.5)
	b. Neutral	3 (13)
	c. Agree	10 (43.5)
22	COVID-19 pandemic inhibits the level up examination process.	
	a. Disagree	16 (69.6)
	b. Neutral	4 (17.4)
	c. Agree	3 (13)
23	National board examination is delayed by the outbreak.	
	a. Disagree	1 (4.3)
	b. Neutral	14 (60.9)
	c. Agree	8 (34.8)

### 3.3 Learning process during the outbreak

Before the outbreak, most (82.6%) residents used textbooks and journals as their primary sources of learning, while during the COVID-19 pandemic, interestingly, 69.5% of residents used online lectures either from the school or Association of Pediatric Surgeons in addition to textbooks and journals. Only 26.1% of participants still used textbooks and journals as the primary sources of learning (Table 3). During the pandemic, we changed the morning reporting of residents from off-line to online meeting. Most residents (82.6%) considered the online morning reports to be good; however, 65.2% of residents thought that off-line morning reports were better than online meeting. About 91.3% of participants agreed that live view surgery was very important to obtain the necessary skills' competence of pediatric surgery during the outbreak. While 82.6% of subjects assumed that live view surgery was important for all cases of elective and emergency surgeries, 78.3% of residents felt that live view surgery was necessary only for interesting cases (Table 3).

Approximately 73.9% of residents expressed that the restriction of resident number in the outpatient clinics during the outbreak did not hamper them from gaining the necessary skills; however, 39.1% of participants said that the restriction of resident number during the ward rounds inhibited them from obtaining the needed skills (Table 3). Accordingly, most residents (82.6%) thought that their competence was declining during the pandemic. Some residents (43.5%) assumed that virtual outpatient and ward round methods are necessary to obtain the necessary skills, particularly for residents who were not on duty in the hospital (Table 3).

### 3.4 Academic evaluation

During the pandemic, 91.3% of participants agreed that they had more time to complete their academic assignments, including thesis completion (43.5%), length of study (43.5%), and level up examination (69.6%). Moreover, 34.8% of participants thought that the pandemic would delay their taking the national board examination (Table 3).

### 3.5 Residents' suggestions to improve the quality of their residency program during the outbreak

There were several suggestions from residents to gain the needed skills and knowledge during the outbreak as follows: 1) virtual didactic methods; 2) maintain and improve the quality of the online learning process; 3) comprehensive scheduling for elective surgeries; and 4) gradually increase the number of elective surgeries.

### 3.6 The impact of the seniority and sex of the participants on the responses to the questionnaire

Interestingly, some responses to the questionnaire were affected by the seniority and sex of the participants, including the compliance level of resident in following the guidelines of personal protective equipment use in the hospital during the pandemic is low (*p* = 0.01), COVID-19 pandemic prolongs the study periods (*p* = 0.024), and COVID-19 pandemic hampers the completion of the thesis (*p* = 0.037); and the frequency of morning report should be increased during the pandemic (*p* = 0.011), the restriction of resident number during the ward rounds inhibits them from obtaining the necessary skills and knowledge (*p* = 0.013), virtual outpatients and ward round method are necessary to obtain the needed skills, particularly for residents who were not on duty in the hospital (*p* = 0.002), COVID-19 pandemic prolongs the study periods (*p* = 0.000), and national board examination is delayed by the outbreak (*p* = 0.012), respectively (Table 4).

**Table 4:** The impact of the seniority and sex of the participants on the responses to the questionnaire.

No	Questions	Resident			*p-*value	Sex		*p*-value
		Senior	Middle	Junior		Male	Female	
	**A. Perspective resident on COVID-19 infection during the residency program**	**N (%)**	**N (%)**	**N (%)**		**N (%)**	**N (%)**	
1	Do you feel worried about getting COVID-19 infection during performing surgeries?							
	a. Yes	9 (100)	6 (100)	8 (100)	N/A	15 (100)	8 (100)	N/A
	b. No	0	0	0		0	0	
	c. Don't know	0	0	0		0	0	
2	In your opinion, do elective pediatric surgical procedures need to be reduced during the COVID-19 pandemic?							
	a. Disagree	2 (22.2)	0	0	0.48	2 (13.3)	0	0.78
	b. Neutral	0	1 (16.7)	1 (12.5)		1 (6.7)	1 (12.5)	
	c. Agree	7 (77.8)	5 (83.3)	7 (87.5)		12 (80.0)	7 (87.5)	
3	During COVID-19, what is the percentage of decrease in the number of the elective pediatric surgeries?							
	a. None	0	0	1 (12.5)	0.15	1 (6.7)	0	0.20
	b. <25%	0	0	3 (37.5)		2 (13.3)	1 (12.5)	
	c. 25-<50%	4 (44.4)	2 (33.3)	3 (37.5)		8 (53.3)	1 (12.5)	
	d. 50-<75%	4 (44.4)	4 (66.7)	1 (12.5)		4 (26.7)	5 (62.5)	
	e. 75-100%	1 (11.2)	0	0		0	1 (12.5)	
4	During COVID-19, what is the percentage of decrease in the number of the emergency pediatric surgeries?							
	a. None	0	0	0	0.05	0	0	0.44
	b. <25%	0	0	4 (50)		3 (20)	1 (12.5)	
	c. 25-<50%	9 (100)	4 (66.7)	3 (37.5)		9 (60)	7 (87.5)	
	d. 50-<75%	0	2 (33.3)	1 (12.5)		3 (20)	0	
	e. 75-100%	0	0	0		0	0	
5	The compliance level of resident in following the guidelines of personal protective equipment use in the hospital during the pandemic is low.							
	a. Disagree	1 (11.1)	3 (50)	7 (87.5)	0.01∗	9 (60.0)	2 (25)	0.25
	b. Neutral	8 (88.9)	3 (50)	0		5 (33.3)	6 (75)	
	c. Agree	0	0	1 (12.5)		1 (16.7)	0	
	**B. Learning process during the outbreak**							
6	Which learning methods did you usually use before COVID-19 outbreak?							
	a. Textbooks and journals	9 (100)	5 (83.3)	5 (62.5)	0.20	13 (86.6)	6 (75)	0.69
	b. Webinar, lectures and workshops held by the school or Indonesian Association of Pediatric Surgeon (PERBANI) or other Pediatric Surgery Association	0	1 (16.7)	2 (25)		1 (6.7)	2 (25)	
	c. Virtual didactic tools (example: Virtual Reality Anatomy, Minimally Invasive Surgery Trainer-Virtual Reality, etc.)	0	0	1 (12.5)		1 (6.7)	0	
	d. Others	0	0	0		0	0	
7	Which learning methods do you usually use during the outbreak?							
	a. Textbooks and journals	3 (33.3)	1 (16.7)	2 (25)	0.16	2 (13.3)	4 (50)	0.13
	b. Webinar, lectures and workshops held by the school or Indonesian Association of Pediatric Surgeon (PERBANI) or other Pediatric Surgery Association	6 (66.7)	4 (66.6)	6 (75)		12 (80)	4 (50)	
	c. Virtual didactic tools (example: Virtual Reality Anatomy, Minimally Invasive Surgery Trainer-Virtual Reality, etc.)	0	1 (16.7)	0		1 (6.7)	0	
	d. Others	0	0	0		0	0	
8	During the pandemic, the online morning report is already good to achieve the skills and knowledge.							
	a. Disagree	0	0	1 (12.5)	0.90	0	1 (12.5)	0.21
	b. Neutral	1 (11.1)	1 (16.7)	1 (12.5)		3 (20)	0	
	c. Agree	8 (88.9)	5 (83.3)	6 (75.)		12 (80)	7 (87.5)	
9	Offline morning report is better than online meeting to achieve the skills and knowledge.							
	a. Disagree	2 (22.2)	1 (16.7)	2 (25)	0.18	3 (20)	2 (25)	0.66
	b. Neutral	0	0	3 (37.5)		3 (20)	0	
	c. Agree	7 (77.8)	5 (83.3)	3 (37.5)		9 (60)	6 (75)	
10	The frequency of morning report should be increased during the pandemic.							
	a. Disagree	0	2 (33.3)	2 (25)	0.15	2 (13.3)	2 (25)	0.011∗
	b. Neutral	7 (77.8)	1 (16.7)	4 (50)		11 (73.4)	1 (12.5)	
	c. Agree	2 (22.2)	3 (50.0)	2 (25)		2 (13.3)	5 (62.5)	
11	Live view surgery method is important to gain the surgical skill during COVID-19 pandemic.							
	a. Disagree	0	0	2 (25)	0.17	1 (6.7)	1 (12.5)	0.59
	b. Neutral	0	0	0		0	0	
	c. Agree	9 (100)	6 (100)	6 (75)		14 (93.3)	7 (87.5)	
12	Live view surgery method is important for all pediatric surgical cases, including elective and emergency cases.							
	a. Disagree	0	0	2 (25)	0.17	1 (6.7)	1 (12.5)	0.78
	b. Neutral	2 (22.2)	0	0		2 (13.3)	0	
	c. Agree	7 (77.8)	6 (100)	6 (75)		12 (80.0)	7 (87.5)	
13	Live view surgery method is important only for interesting pediatric surgical cases.							
	a. Disagree	3 (33.3)	1 (16.7)	1 (12.5)	0.69	4 (26.7)	1 (12.5)	0.62
	b. Neutral	0	0	0		0	0	
	c. Agree	6 (66.7)	5 (83.3)	7 (87.5)		11 (73.3)	7 (87.5)	
14	The restriction of resident number in the outpatients' clinics during the outbreak hampers them from gaining the necessary skills and knowledge.							
	a. Disagree	5 (55.6)	4 (66.7)	8 (100)	0.12	10 (66.7)	7 (87.5)	0.80
	b. Neutral	2 (22.2)	2 (33.3)	0		3 (20.0)	1 (12.5)	
	c. Agree	2 (22.2)	0	0		2 (13.3)	0	
15	The restriction of resident number during the ward rounds inhibits them from obtaining the necessary skills and knowledge.							
	a. Disagree	3 (33.3)	3 (50)	4 (50)	0.45	3 (20)	7 (87.5)	0.013∗
	b. Neutral	2 (22.2)	2 (33.3)	0		4 (26.7)	0	
	c. Agree	4 (44.5)	1 (16.7)	4 (50)		8 (53.3)	1 (12.5)	
16	Virtual outpatients and ward round method are necessary to obtain the needed skills, particularly for residents who were not on duty in the hospital.							
	a. Disagree	3 (33.3)	3 (50)	3 (37.5)	0.39	2 (13.3)	7 (87.5)	0.002∗
	b. Neutral	2 (22.2)	2 (33.3)	0		4 (26.7)	0	
	c. Agree	4 (44.5)	1 (16.7)	5 (62.5)		9 (60)	1 (12.5)	
17	The competence gain is declining during the pandemic.							
	a. Disagree	1 (11.1)	0	1 (12.5)	0.56	1 (6.7)	1 (12.5)	0.78
	b. Neutral	2 (22.2)	0	0		2 (13.3)	0	
	c. Agree	6 (66.7)	6 (100)	7 (87.5)		12 (80.0)	7 (87.5)	
	**C. Academic evaluation**							
18	During COVID-19 pandemic, you have more time to finish the academic assignments.							
	a. Disagree	0	0	1 (12.5)	0.57	0	1 (12.5)	0.59
	b. Neutral	0	0	1 (12.5)		1 (6.7)	0	
	c. Agree	9 (100)	6 (100)	6 (75)		14 (93.3)	7 (87.5)	
19	The completion of the academic assignments can be achieved on time.							
	a. Disagree	0	0	1 (12.5)	0.41	0	1 (12.5)	0.43
	b. Neutral	2 (22.2)	0	0		2 (13.3)	0	
	c. Agree	7 (77.8)	6 (100)	7 (87.5)		13 (86.7)	7 (87.5)	
20	COVID-19 pandemic prolongs the study periods.							
	a. Disagree	3 (33.3)	5 (83.3)	2 (25.0)	0.024∗	2 (13.3)	8 (100)	0.000∗
	b. Neutral	5 (55.6)	0	1 (12.5)		6 (40.0)	0	
	c. Agree	1 (11.1)	1 (16.7)	5 (62.5)		7 (46.7)	0	
21	COVID-19 pandemic hampers the completion of the thesis.							
	a. Disagree	4 (44.4)	0	6 (75.0)	0.037∗	9 (60)	1 (12.5)	0.07
	b. Neutral	2 (22.2)	1 (16.7)	0		2 (13.3)	1 (12.5)	
	c. Agree	3 (33.4)	5 (83.3)	2 (25.0)		4 (26.7)	6 (75.0)	
22	COVID-19 pandemic inhibits the level up examination process.							
	a. Disagree	7 (77.8)	3 (50)	6 (75)	0.80	11 (73.3)	5 (62.5)	0.64
	b. Neutral	1 (11.1)	2 (33.3)	1 (12.5)		3 (20)	1 (12.5)	
	c. Agree	1 (11.1)	1 (16.7)	1 (12.5)		1 (6.7)	2 (25)	
23	National board examination is delayed by the outbreak.							
	a. Disagree	1 (11.1)	0	0	0.22	1 (6.7)	0	0.012∗
	b. Neutral	6 (66.7)	5 (83.3)	3 (37.5)		6 (40.0)	8 (100)	
	c. Agree	2 (22.2)	1 (16.7)	5 (62.5)		8 (53.3)	0	

N/A, not applicable; ∗, *p* < 0.05.

## 4 Discussion

Here, we show that the pediatric surgery residency program at our institution has been significantly affected by the COVID-19 pandemic. Our findings further confirmed previous reports [2, 3]. However, there are several novelties of our study: 1) pediatric surgery residency program (*vs.* plastic surgery training [2]); 2) developing country (vs. developed countries [2,3,4]); 3) prospective design using questionnaire (*vs.* retrospective design [3]); and 4) comprehensively developed the questionnaire into four aspects that might affect the residency program: a) the perspectives of residents about COVID-19 infection; b) learning process; c) academic evaluations; and d) residents' suggestions for residency program improvement (*vs.* general questionnaire [2] or authors' perspective [4]).

All residents are worried they will become infected by COVID-19 during their residency program in the hospital. Accordingly, they agreed that elective surgeries should be postponed during the pandemic. When compared with the importance of the residents safeguarding the well-being of their families from the possibility of getting cross-infected by COVID-19 due to the residents' potential exposure at the hospital, the training program was no longer considered as important anymore for the residents [3].

Moreover, since the COVID-19 pandemic, we have shifted our morning report from off-line to online meetings. Most residents are satisfied with the changes, although some residents thought that offline morning report was better to gain skills and knowledge than online meetings (Table 3). Some residents agreed that the skills and knowledge can be achieved by both offline and online morning report. It does not mean that they do not have a preference in the reporting style. However, they believed that the changes in the morning report method is to adapt to the pandemic and the results are good, while the offline morning report is still considered good too.

We also evaluated the learning process regarding the competence gained during the pandemic. Our data showed that most residents believe that their competence is declining during the pandemic. This finding might be associated with the fact that almost all elective surgical cases were postponed during the pandemic. Postponing elective surgeries occurred in every country affected by the COVID-19, including the USA [3] and Australia [7]. Most residents agreed that live view surgery with virtual outpatient services and ward rounds will be useful to solve this challenge.

While there are limited activities to gain the skills of pediatric surgery during the pandemic, intriguingly, most residents claimed that this outbreak gave them more time to finish their academic assignments, including extended study time for level up examinations and completion of their thesis. These advantages might be related to the policy that restricted the number of residents during the outpatient services, ward rounds and surgical procedures. As a result, most of them stayed at home, and only a limited number of rotating residents (*i.e*., three per round) were allowed to perform residency tasks each day in the hospital.

Interestingly, some responses to questionnaire are affected by the seniority and sex of participants. For instance, 87.5% of junior and 46.7% of male residents believed that the COVID-19 pandemic prolongs the study periods, while 55.6% of senior and all female residents were neutral and disagreed to the statement. A qualitative study is important to further elaborate their thought.

One of the residents' suggestions was to gradually increase the number of elective surgeries. This response reflects that it is not clear whether the COVID-19 pandemic will end in a few months or even in the next few years, and as a proper response, our government declared a “new normal” policy on June 1, 2020 to begin the adaptations of the daily activities to the COVID-19 pandemic, involving clinical and surgical services [8]. Our pediatric surgery services have adapted our scheduling practices as well starting on June 8, 2020, when we began to perform a) only one major surgery every week on Tuesdays; and b) two or three minor procedures on another working day each week [9].

Although the response rate of our study was 100%, a relatively small number of pediatric surgery residents involved in this study should be taken into consideration during interpretation of our findings. Another limitation of our study was limited to the subjective opinion of the residents without objective support.

## 5 Conclusions

The pandemic COVID-19 has had a significant impact on the development of pediatric surgery residency programs. Moreover, the responses to the questionnaire are affected by the seniority and sex of the residents. A comprehensive approach is needed to maintain the high standard of competence of pediatric surgery without compromising our safety from the risk of COVID-19 infection.

## Declarations

### Author contribution statement

Gunadi: Conceived and designed the experiments; Analyzed and interpreted the data; Wrote the paper.

Eko Purnomo, Andi Dwihantoro, Nunik Agustriani and Akhmad Makhmudi: Conceived and designed the experiments; Wrote the paper.

Naisya Balela and Alvin Santoso Kalim: Performed the experiments; Analyzed and interpreted the data.

William Widitjiarso, Fadil Fahri and Audric Kenny Tedja: Performed the experiments.

### Funding statement

This work was supported by the National Agency for Research and Innovation, Indonesia.

### Data availability statement

Data included in article/supplementary material/referenced in article.

### Declaration of interests statement

The authors declare no conflict of interest.

### Additional information

No additional information is available for this paper.
